# Epidermal Growth Factor Receptor (EGFR) Amplification May Lead to Invalid Cobas EGFR Mutation Test v2 Results

**DOI:** 10.3390/diagnostics15080948

**Published:** 2025-04-08

**Authors:** Min-Shu Hsieh, Tze-Chun Hung, Hsien-Neng Huang, Chao-Wen Lu, Hsiang-Wei Hu, Jin-Yao Lai, Wen-Yao Lee, Jin-Shing Chen

**Affiliations:** 1 Department of Pathology, National Taiwan University Hospital, Taipei 10845, Taiwan; mshsieh065@gmail.com (M.-S.H.); 108368@ntuh.gov.tw (T.-C.H.); hsiangweihu@ntuh.gov.tw (H.-W.H.); jennifer321kimo@gmail.com (J.-Y.L.); 2 Department of Pathology, National Taiwan University Cancer Center, Taipei 10672, Taiwan; 3 Graduate Institute of Pathology, National Taiwan University College of Medicine, Taipei 10845, Taiwan; ken102huang@gmail.com (H.-N.H.); i2363160@gmail.com (C.-W.L.); 4 Department of Pathology, National Taiwan University Hospital Hsin-Chu Branch, Hsinchu 30261, Taiwan; 5 Division of Thoracic Surgery, Department of Surgery, National Taiwan University Hospital, and National Taiwan University College of Medicine, Taipei 10845, Taiwan; chenjs.ntu@gmail.com; 6 Division of Thoracic Surgery, Department of Surgery, Fu Jen Catholic University Hospital, New Taipei City 24205, Taiwan

**Keywords:** non-small cell lung cancer, EGFR mutation, cobas EGFR test v2, invalid test results, internal control Ct values, EGFR amplification

## Abstract

**Background/Objectives:** The cobas EGFR test v2, used for detecting EGFR mutations, can yield invalid results due to internal control (IC) issues, such as “IC not detected”, “IC out of range: high Ct value”, or “IC out of range: low Ct value”. This study aimed to examine the incidence of invalid cobas results and explored the mechanism behind low IC Ct values. **Methods:** We retrospectively reviewed invalid cases, linking undetectable or high IC Ct values to inadequate DNA from small biopsies, as determined by conducting a pathological review. Cases with low IC Ct values were further tested, with the hypothesis of EGFR amplification confirmed using Sanger sequencing, the Idylla assay, next-generation sequencing (NGS), and fluorescence in situ hybridization (FISH). **Results:** Among 4148 cases, the incidence of invalid results was 0.99% (41/4148). In four cases with low IC Ct values, EGFR amplification was confirmed using alternative methods with successful cobas testing on diluted DNA. **Conclusions:** These findings suggest that EGFR amplification, rather than specimen inadequacy, is the cause of low IC Ct results, making rebiopsy unnecessary. Alternative assays or diluted DNA allow for successful EGFR testing.

## 1. Introduction

Lung cancer is a leading cause of death worldwide, with non-small cell lung cancer (NSCLC) making up 80% to 85% of all deaths, mainly comprising adenocarcinoma (ADC), squamous cell carcinoma (SCC), and other rare subtypes [1,2].

In Taiwan, most patients with lung cancer have stage III or IV disease at the time of diagnosis [2,3,4]. Owing to the initiation of several health policies, including smoking cessation, precision therapy, and low-dose computed tomography (LDCT) since 1997, a significant diagnostic shift from late to early-stage lung cancer has been observed in the National Taiwan University Hospital (NTUH) [5]. However, a considerable proportion of advanced NSCLC still requires systemic treatment. The National Comprehensive Cancer Network (NCCN) Guidelines for NSCLC recommend biomarker testing, including EGFR, ALK, ROS1, KRAS, MET, RET, NTRK, BRAF, and ERBB2, for actionable oncogenic driver mutations [6,7].

In East Asia, EGFR is the most common driver mutation, with a prevalence ranging from 20% to 76% across different studies [2,3,5]. Exon 21 L858R point mutation and Exon 19 deletion are the most common EGFR mutations, usually referred to as classic EGFR mutations, which are generally sensitive to all three generations of EGFR tyrosine kinase inhibitors (EGFR-TKIs) [2,4,5]. Therefore, the accurate and timely detection of EGFR mutations is crucial for the treatment of advanced NSCLC. Various molecular diagnostic techniques are employed, including Sanger sequencing, real-time polymerase chain reaction (RT-PCR)-based assays, droplet digital PCR, mass spectrometry, and next-generation sequencing (NGS). Each method has its own limitations, including sensitivity and specificity, turnaround times, technique requirements, and cost.

Upfront next-generation sequencing (NGS) for patients with metastatic NSCLC is considered a cost-saving measure in the United States, whereas exclusionary testing for EGFR is considered the most cost-effective method in East Asia, including Taiwan [8,9,10]. In Taiwan, the EGFR test is typically conducted using real-time polymerase chain reaction (RT-PCR)-based methods, such as the Roche cobas EGFR mutation test v2 (Roche Molecular Systems, Inc., Pleasanton, CA, USA) and the Idylla EGFR mutation test (Biocartis, Mechelen, Belgium).

The cobas EGFR mutation test v2 is the most commonly used test platform in Taiwan. Despite RT-PCR-based EGFR tests being convenient and more sensitive than Sanger sequencing, they have certain limitations. For example, they can only detect a limited number of mutation hotspots. In addition, false-positive reports of Exon 20 insertion (ex20ins) may occur in patients with EGFR amplification, and false-negative reports of Exon 21 L858R may occur in those with L858R doublet mutations [11,12,13,14]. Occasionally, the cobas test may yield invalid results when the DNA samples do not meet the criteria set for the internal control (IC). Most cases with invalid codes, such as “Internal Control could not be detected”, were small biopsies, indicating an inadequate amount of EGFR DNA. In our department, some specimens reported as invalid due to “Internal Control (IC) out of range: low cycle threshold (Ct)” were actually surgically resected lymph nodes, which should have been adequate for the cobas test. Since EGFR Exon 28 is used as the IC by the cobas test, a sample with a low IC Ct value implies increased EGFR copy numbers, such as high polysomy or amplification. To confirm the hypothesis that EGFR amplification may lead to an invalid report in the cobas test, we retrospectively reviewed all specimens reported as “invalid” by the cobas test in our department, focusing on cases with the code “IC out of range: low Ct”.

## 2. Materials and Methods

### 2.1. Case Selection

**Figure 1:**
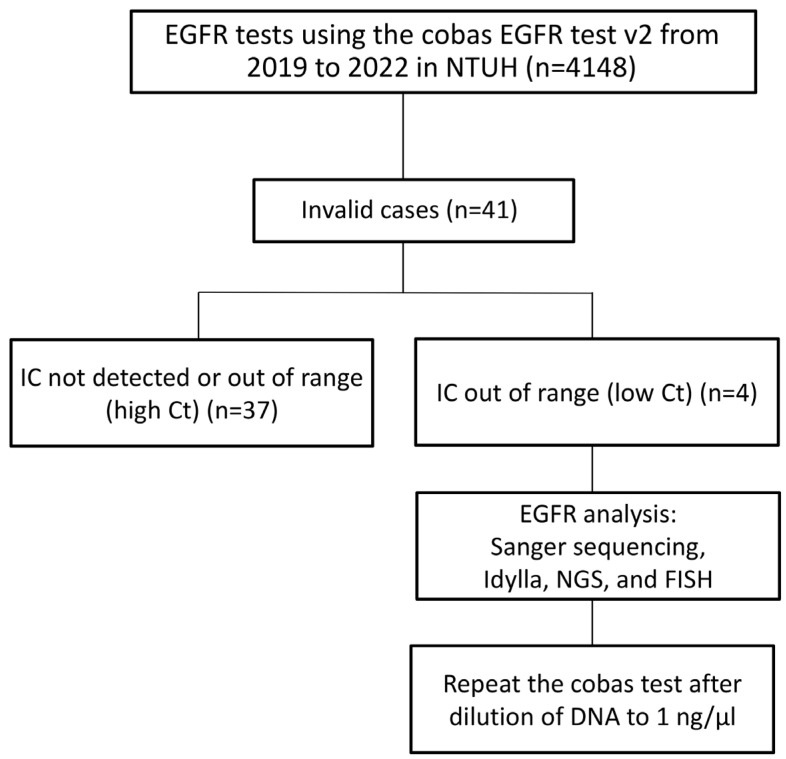
Study algorithm: Among 41 cases reported as “invalid” by the cobas EGFR test v2, four cases with “Internal Control (IC) out of range: low cycle threshold (Ct)” were analyzed via Sanger sequencing, Idylla, EGFR fluorescence in situ hybridization (FISH), and next-generation sequencing (NGS), followed by a second-round cobas test after dilution of the DNA sample.

Retrospectively, specimens submitted for the cobas EGFR test v2 from May 2019 to December 2022 in the Department of Pathology NTUH were reviewed. Among 4148 samples during this period, 41 were reported as “invalid” by the cobas test. For each case, the invalid code reported by the cobas test was retrieved. Clinical information was collected from medical records. All available HE-stained slides were reviewed by two thoracic pathologists (Min-Shu Hsieh and Hsiang-Wei Hu). The specimen types, histologic patterns, and tumor contents were recorded. This study was approved by the Research Ethics Committee of the National Taiwan University Hospital (202302134RINC). The study’s algorithm is summarized in Figure 1.

### 2.2. Mutation Analysis

For the cobas EGFR mutation test v2 (Roche Molecular Systems), DNA was extracted from formalin-fixed paraffin-embedded (FFPE) specimens using the cobas DNA Sample Preparation Kit (Roche, Tucson, AZ, USA). The extracted DNA sample was diluted to 2 ng/μL, and then amplified using the cobas z480 system according to the manufacturer’s instructions. For cases with the invalid code “Internal Control (IC) out of range: low cycle threshold (Ct)”, the extracted DNA concentration was further diluted into 1 ng/μL for the second-round cobas EGFR test.

For Sanger sequencing, DNA was extracted from FFPE specimens using a DNA Extraction Kit (Qiagen, Hilden, Germany) and subjected to PCR (Biometra Thermocycler TProfessional Basic 96, Montreal Biotech, Dorval, QC, Canada) using specific primers (Supplementary Table S1). These PCR amplicons were purified using ExoSAP-IT (Thermo Fisher Scientific, Waltham, MA, USA) and cycle-sequenced using ABI BigDye V3.1 with an ABI 3730xl genetic analyzer (Applied Biosystems, Waltham, MA, USA). The sequences were compared with the GenBank-archived human EGFR sequences.

For Idylla EGFR mutation testing, 5 µm FFPE tissue sections were placed into the Idylla EGFR mutation test cartridge and submitted to the fully automated Idylla platform (Biocartis). After 150 min, the final report was released directly from the system following the automatic onboard post-PCR curve analysis.

For NGS, the Archer VariantPlex solid tumor focus v2 panel (ArcherDX, Boulder, CO, USA), a targeted enrichment method using anchored multiplex PCR (AMP), was used. The panel enabled the detection of single-nucleotide variants (SNVs) in 20 target genes and the analysis of copy number variation (CNV) for 12 genes, including EGFR. DNA quality was assessed using the Archer PreSeq DNA QC assay (ArcherDX), and then used to create the libraries according to the manufacturer’s instructions. For each patient, 100 ng of total DNA was fragmented and amplified using specific primers provided by the manufacturer. Libraries were quantified using the KAPA library quantification kit (Roche), and then pooled to achieve an equimolar concentration. Next-generation sequencing (NGS) was performed on the NovaSeq X Plus Platform (Illumina, San Diego, CA, USA), and the results were analyzed using the Archer Analysis v7.3.2 software (ArcherDX).

### 2.3. Epidermal Growth Factor Receptor (EGFR) Fluorescence In Situ Hybridization (FISH)

EGFR FISH was performed using a ZytoLight SPEC EGFR/CEN 7 dual-color probe (ZytoVision GmbH, Bremerhaven, Germany) for cases with low IC Ct values reported by the cobas test. In each case, 50 non-overlapping tumor cell nuclei were evaluated (by Tze-Chun Hung and Min-Shu Hsieh) according to the Colorado Scoring Criteria: disomy (score = 1), low trisomy (score = 2), high trisomy (score = 3), low polysomy (score = 4), high polysomy (score = 5, defined as ≥40% of cells displaying ≥ 4 copies of the EGFR signal), and gene amplification (score = 6, defined as (a) an EGFR-to-CEP7 ratio ≥ 2 overall scored nuclei and calculated using the sum of EGFR divided by the sum of CEP7 when the mean CEP7 per cell is ≥2 copies, (b) the presence of gene cluster (≥4 spots) in ≥10% of tumor cells, or (c) at least 15 copies of the EGFR signals in ≥10% of tumor cells) [15].

## 3. Results

### 3.1. The Invalid Rate Is 0.99% Among 4148 Cobas Epidermal Growth Factor Receptor (EGFR) Tests Conducted at the National Taiwan University Hospital (NTUH) from 2019 to 2022

Among 4148 EGFR tests using the cobas EGFR mutation test v2 from January 2019 to September 2022 in the Department of Pathology at the NTUH, there were 4107 valid and 41 invalid test reports. The invalid rate was 0.99% (Table 1).

The invalid codes of all 41 cases with “invalid” reports are summarized in Table 2. According to the description of codes by the manufacturer, the cases with invalid reports were due to “Internal control out of range: low Ct value (4/41, 9.8%)”, “Internal control out of range: high Ct value (26/41, 63.4%)”, and “Internal control could not be detected (11/41, 26.8%)”. Therefore, these 41 cases could be further categorized into two groups: those whose internal control had a high Ct value or could not be detected, representing cases with inadequate specimens or poor DNA quality, and those whose internal control had a low Ct value, representing cases with extremely high EGFR DNA content. Except for four cases with the invalid code R814, the majority of cases (90.2%) with invalid cobas tests were due to inadequate specimens, leading to high or undetectable IC Ct values.

### 3.2. Epidermal Growth Factor Receptor (EGFR) Amplification Resulted in a Low Cycle Threshold (Ct) Value for the Internal Control in the Four Invalid Cases, Leading to Invalid Results in the Cobas EGFR Test v2

**Figure 2:**
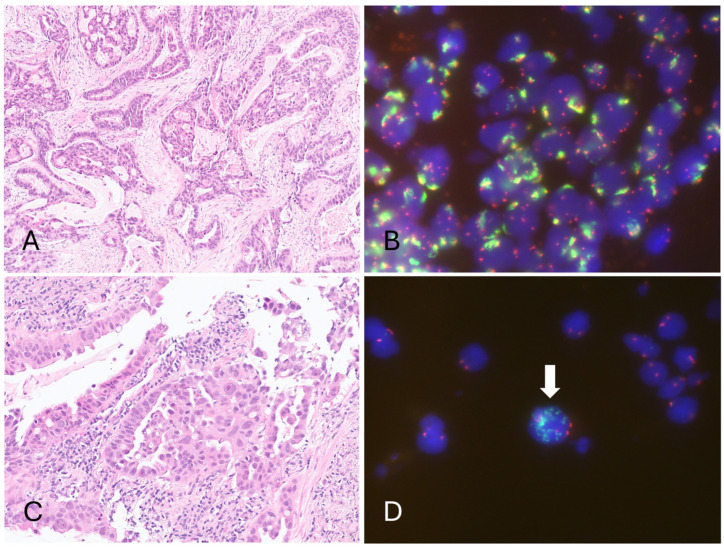
Pathological findings and EGFR FISH of cases with invalid cobas EGFR test results (R814, low Ct). (**A**) Case 1 adenocarcinoma with complex glandular structures. (**B**) FISH revealing a large EGFR gene cluster in tumor cells. (**C**) Case 2 with micropapillary patterns. (**D**) FISH revealing more than 15 copies of EGFR signals in the tumor cell (arrow). (Hematoxylin and eosin stain, magnification: (**A**) 100×, (**C**) 200×).

**Figure 3:**
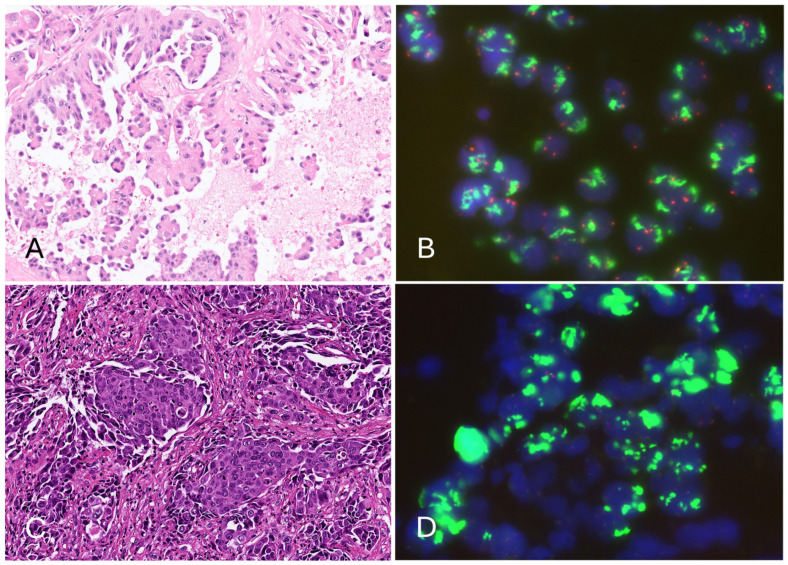
Pathological findings and EGFR FISH of cases with invalid cobas EGFR test results (R814, low Ct). (**A**) Case 3 adenocarcinoma with micropapillary structures. (**B**) FISH revealing a large EGFR gene cluster in tumor cells. (**C**) Case 4 adenocarcinoma arranged in solid nests. (**D**) FISH revealing giant EGFR gene cluster in tumor cells. (Hematoxylin and eosin stain, magnification: (**A**,**C**) 200×).

All cases with a high or undetectable IC Ct value were small biopsies, suggesting that rebiopsy for larger specimens is necessary for the cobas EGFR test. On the contrary, two of four invalid cases with a low IC Ct value were large specimens, such as surgically removed lymph nodes, and all these cases had tumor contents of at least 15%. For the four invalid cases with a low IC Ct value, their clinical characteristics and pathological/molecular findings are summarized in Table 3 and Table 4, respectively. All four cases were male, and their ages ranged from 54 to 72 years. Three of them were smokers. The size of the primary tumor ranged from 2.8 to 10 cm. One was stage IIIB, and the others were stage IV at the time of diagnosis. Microscopically, all four cases demonstrated high-grade histologic patterns, including complex glandular structures and micropapillary or solid patterns (Figure 2 and Figure 3). The out-of-range low IC Ct values of these specimens, as reported by the cobas test, suggest that these tumor cells might have unusually high EGFR amplification values, which exceeds the normal range of most NSCLC specimens. This hypothesis was confirmed using EGFR FISH, which revealed EGFR amplification (based on the definition of Colorado Scoring Criteria) in all cases with a low IC Ct value, including three with large EGFR gene clusters and one with at least 15 copies of the EGFR signals in ≥10% of tumor cells (Figure 2 and Figure 3). Furthermore, Sanger sequencing and the Idylla EGFR test were successfully performed in all four cobas-invalid cases. Three cases had sufficient tissue for NGS. The EGFR mutations in two cases (one L858R and one Ex19del) were all detected by Sanger sequencing, the Idylla test, and NGS. NGS also reported EGFR amplification in all tested cases. Notably, Idylla also reported an aberrant T790M mutation result in case 4, which was not detected by Sanger sequencing or NGS (Table 4). The IC Ct value reported by the Idylla was also remarkably low (14.8~17.9) as the usual IC Ct value of Idylla ranges from approximately 20 to 24. These findings indicate that cases with invalid cobas test results coded as R814 do not require rebiopsy, as mutation testing can be successfully performed using alternative methods.

### 3.3. All Four Cases with a Low Internal Control (IC) Cycle Threshold (Ct) Value Are Valid in the Second Round of Cobas Epidermal Growth Factor Receptor (EGFR) Testing Using Diluted Deoxyribonucleic Acid (DNA) Specimens

Since invalid cobas tests with a low IC Ct value were attributed to EGFR amplification rather than low tumor content or poor DNA quality, we hypothesized that it might be feasible to use diluted DNA samples (from 2 ng/μL to 1 ng/μL) for the second round of cobas EGFR testing. Second-round tests using diluted DNA specimens were valid in all four cases, and EGFR mutations identified by other methods, such as Sanger sequencing, Idylla, or NGS, were also detected by the cobas test (Table 4). However, aberrant ex20ins mutations, which were undetectable by Sanger sequencing, Idylla, or NGS, were reported in all four cases (Table 4).

## 4. Discussion

The Roche cobas EGFR mutation test v2 (Roche), a companion diagnostic tool for EGFR-TKI therapy approved by the U.S. Food and Drug Administration, is widely used by pathology laboratories in Taiwan. This study demonstrated that the cobas EGFR test v2 has a high success rate, with only 0.99% of results being invalid out of more than 4000 tests. Cases with invalid results can be categorized into two distinct groups: those with insufficient DNA samples, reflected by undetectable (0.26%) or excessively high IC Ct values (0.63%), and those with EGFR amplification, leading to abnormally low IC Ct values (0.10%).

**Figure 4:**
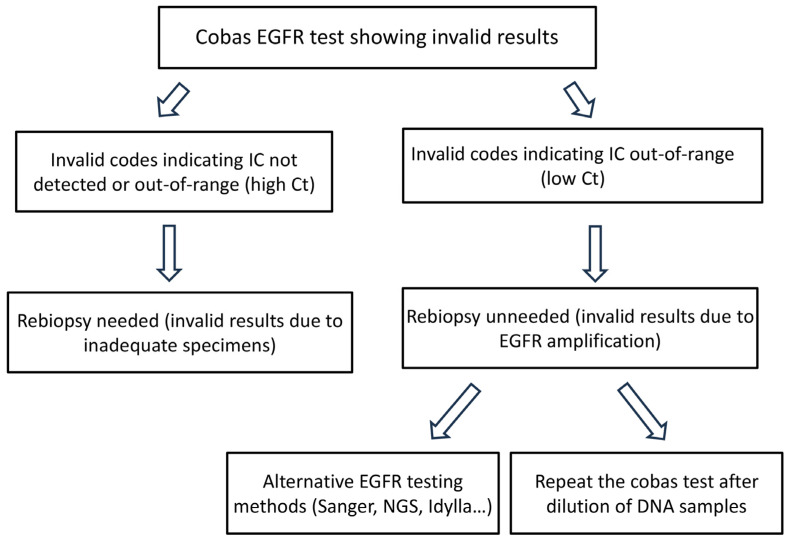
Flow diagram for invalid cobas EGFR test results.

In cases of insufficient or inadequate DNA samples, which were all obtained from small biopsies, a rebiopsy may be performed to obtain adequate tissue specimens or by using more sensitive tests, such as NGS. In contrast, cases with EGFR amplification, characterized by low IC Ct values, actually had sufficient tissue specimens with adequate DNA samples. In these instances, mutation analysis can be successfully performed using alternative methods, such as the Idylla assay, NGS, and Sanger sequencing, or by conducting a second-round cobas test with diluted DNA samples. Based on the above findings, a flow diagram is proposed for cases with invalid cobas test results (Figure 4).

Generally, in our hospital, the time from the initial diagnosis of lung cancer to the EGFR test report spans about one to two weeks. However, EGFR amplification leading to invalid cobas EGFR test results may extend the time from initial diagnosis to the initiation of systemic therapy. In the four cases from this study, it took two weeks to three months to obtain the final EGFR results via NGS or Sanger sequencing after the initial invalid cobas tests. It is important to note that the R814 “Internal Control out of range (Low Ct)” invalid code in the cobas test indicates the probable presence of EGFR amplification rather than an inadequate tissue specimen.

RT-PCR-based methods, such as the cobas EGFR test v2 and the Idylla EGFR mutation test, are more sensitive than Sanger sequencing, with a limit of detection around 5% [16,17]. Both the cobas and Idylla tests are convenient and commonly used single-gene tests. However, the cobas EGFR test may produce false-negative or false-positive results. Since RT-PCR methods rely on primers to detect prevalent EGFR mutations, they may miss rare mutations. For example, it is estimated that more than 40% of NSCLC cases with EGFR Exon 20 insertions (ex20ins) may be undetected by the cobas EGFR test compared to NGS-based genetic testing [18,19]. Additionally, the primers designed for the cobas test may be affected by complex genetic alterations, such as EGFR L858R-K860I and L858R-L861F doublet mutations, which can lead to false-negative results for the L858R mutation [12,13]. Onozawa et al. reported a case of EGFR L858R-K860I cis doublet mutation, where the corresponding L858R mutation (c.2573 T > G) was not detected by the cobas EGFR mutation test v2 [13]. In our hospital, three cases with L858R doublet mutations (two with EGFR L858R-K860I and one with L858R-L861F doublet mutations) were identified by NGS after initially yielding negative results from the cobas EGFR mutation test v2 [12].

EGFR amplification in lung adenocarcinoma can be a de novo event or a secondary acquired resistant mechanism after targeted therapy [20,21]. Sholl et al. analyzed women who did not smoke and who were of East Asian origin with lung adenocarcinomas and found that EGFR amplification was associated with solid histology, advanced clinical stage, and a worse outcome in univariate analysis [20]. EGFR protein overexpression was also significantly more commonly observed in adenocarcinomas with EGFR amplification [21]. They also reported that EGFR amplification was heterogeneously distributed across different histologic patterns, as examined by chromogenic in situ hybridization, and was most prevalent in regions with solid histology [20]. In our study, all four cases with EGFR amplification exhibited high-grade histologic features, including micropapillary, complex glandular structures, solid nests, and tumor necrosis. In our previous analysis of lung adenocarcinoma with EGFR Exon 20 insertion mutations, cases with EGFR amplification were significantly associated with high-grade histologic features [11]. EGFR amplification is considered an on-target acquired resistant mechanism of lung adenocarcinoma treated with EGFR tyrosine kinase inhibitors [21]. It has been reported that sequential liquid biopsies have disclosed the emergence of subclone cells with EGFR amplification in patients treated with osimertinib, suggesting that EGFR amplification is a mechanism of acquired resistance [22,23,24]. EGFR amplification was observed in 5.3% of patients who developed resistance during first-line osimertinib treatment [25]. In patients with EGFR T790M–positive lung cancer who developed acquired resistance to osimertinib, EGFR amplification was observed in those with a retained T790M mutation (9/19, 47.4%) [26,27].

Although EGFR amplification is not an independent prognostic factor, high-level EGFR amplification may lead to aberrant cobas EGFR test results, including invalid tests or false-positive ex20ins. These aberrant cobas results only occur with high EGFR amplification, as disclosed by NGS (usually EGFR copy number > 15). In our department, all cases with cobas-invalid results and low IC Ct values are submitted for alternative mutation tests, such as Sanger sequencing or Idylla, and cases reported as ex20ins by the cobas results are validated by Sanger sequencing to avoid false-positive reports.

In this study, despite the success of a second-round cobas test using diluted DNA samples, all four cases with EGFR amplification and low IC Ct values produced false-positive reports of Exon 20 insertions by the cobas test. These were not detected by other testing methods such as Sanger sequencing, Idylla, or NGS. False positives for Exon 20 insertions using the cobas EGFR test were previously reported by Kanaoka et al., although the underlying mechanism was not discussed [13]. Our study group independently reported cases with aberrant EGFR ex20ins results from the cobas test, and most of these cases also exhibited EGFR amplification [11]. The false positives for EGFR ex20ins in this study further support the association between aberrant ex20ins reports and EGFR amplification when using the cobas EGFR test.

It is worth noting that while the Idylla test was successfully performed in all four cobas-invalid cases with low IC Ct values, an aberrant Exon 20 T790M mutation, undetectable by NGS, was reported in case 4, which had the highest EGFR copy number gain by NGS and largest gene clusters by FISH. This study raises the possibility that RT-PCR-based testing platforms may yield false-positive results when the target gene is highly amplified in the tested samples.

Lastly, this study has some limitations. First, the exact cutoff thresholds for IC Ct values and the primer design of the cobas EGFR test are not publicly available, making it difficult to determine the extent of EGFR amplification that would lead to an invalid report. Second, although the second-round cobas test using diluted DNA specimens from cases with low IC Ct values was successful, this approach does not adhere to the manufacturer’s guidelines and should be regarded as an off-label use of this companion diagnostic tool. Furthermore, the aberrant ex20ins report in these cases still requires validation tests using other testing platforms, such as Sanger sequencing or NGS. Lastly, while EGFR FISH is effective in detecting amplification, it is a relatively qualitative test that does not quantify the EGFR copy number gain. This limitation makes it challenging to distinguish between cases with low-level and high-level EGFR amplification, which may have clinical significance in predicting responses to targeted therapies. Moreover, the exact EGFR amplification level causing invalid cobas test results remains unclear.

## 5. Conclusions

An inadequate specimen with a high or undetectable IC Ct value or a specimen with high EGFR amplification and a low IC Ct value can lead to invalid cobas EGFR tests. The former requires rebiopsy, while the latter can be successfully tested using different platforms or by performing a second-round cobas test with diluted DNA samples. Recognizing the underlying mechanisms that lead to invalid cobas tests can ensure that patients receive timely and accurate treatment.

## Figures and Tables

**Table 1:** Cases reported as “invalid cases” by the cobas EGFR test v2 between 2019 and 2022 at the NTUH.

Year	Cobas Test “Valid”	Cobas Test “Invalid”	Prevalence
2019	718	14	1.91%
2020	1184	10	0.84%
2021	1373	6	0.44%
2022	832	11	1.30%
Total	4107	41	0.99%

**Table 2:** Invalid codes of 41 cases reported as “invalid” by the cobas EGFR test v2.

Invalid Code	Description	Case: Total (Each Code)
R812/R832/R852	Internal Control could not be detected	11 (8/1/2)
R813/R834/R853	Internal Control out of range (High Ct)	26 (24/1/1)
R814	Internal Control out of range (Low Ct)	4
Total		41

**Table 3:** Patient characteristics of four invalid cases.

	Age (Years)	Sex	Smoking	Tumor Site	Tumor Size (cm)	TNM (AJCC 8th)
Case 1	59	Male	Smoker	RUL	7.5	cT4N2M0 (stage IIIB)
Case 2	72	Male	Smoker	LUL	4.4	cT4N3M1c (stage IVB)
Case 3	72	Male	Non-smoker	RLL	2.8	cT4N3M1c (stage IVB)
Case 4	54	Male	Smoker	LUL	10	cT4N3M1a (stage IVA)

Abbreviations: RUL, right upper lobe; LUL, left upper lobe; and RLL, right lower lobe.

**Table 4:** Results of different mutation analysis methods for four cases with invalid code R814 using the cobas EGFR test v2.

	Pathology	Tumor%	First Cobas Test	Sanger	Idylla	EGFR FISH	Second Cobas Test After Dilution of DNA Samples	NGS		
								SNV and InDels	EGFR CNV	MS
Case 1	ADC (acinar, high-grade)	30%	R814	L858R	L858R (IC Ct 16.2)	Amp (large gene clusters)	L858R + Ex20ins (Ct: 31.16)	EGFR (p.L858R) NRAS (p.D176Y) TP53 (p.D281E)	Amp (27.33)	MS-Stable
Case 2	ADC (micropapillary)	15%	R814	WT	WT (IC Ct 17.9)	Amp (>15/cell)	Ex20ins (Ct: 31.01)	N/A	N/A	N/A
Case 3	ADC (micropapillary)	30%	R814	Ex19Del	Ex19Del (IC Ct 16.2)	Amp (large gene clusters)	Ex19Del + Ex20ins (Ct: 31.6)	EGFR ex19del (p.E746_ A750del)	Amp (16.75)	MS-Stable
Case 4	ADC (solid)	25%	R814	WT	T790M (IC Ct 14.8)	Amp (large gene clusters)	Ex20ins (Ct: 31.65)	TP53 (p.P153fs*28)	Amp (28.09)	MS-Stable

Abbreviations: ADC, adenocarcinoma; Amp, amplification; CNV, copy number variation; Ct, cycle threshold; EGFR, epidermal growth factor receptor; Ex, exon; FISH, fluorescence in situ hybridization; IC, internal control; InDels, insertion–deletion; MS, microsatellite status; NGS, next-generation sequencing; NRAS, NRAS proto-oncogene, GTPase; SNV, single-nucleotide variant; TP53, tumor protein p53; and WT, wild type.

## Data Availability

Due to patient privacy concerns and institutional regulations, the data from this study are not publicly available. However, relevant data may be obtained from the corresponding author upon reasonable request.

## Supplementary Materials

The following supporting information can be downloaded at: https://www.mdpi.com/article/10.3390/diagnostics15080948/s1, Table S1: Specific EGFR primers used for Sanger sequencing.

## Abbreviations

The following abbreviations are used in this manuscript:

NSCLC	Non-small cell lung cancer
EGFR	Epidermal Growth Factor Receptor
IC	Internal Control
NGS	Next-generation sequencing
FISH	Fluorescence In Situ Hybridization
