# Supplementary material for: Epidermal Growth Factor Receptor (EGFR) Amplification May Lead to Invalid Cobas EGFR Mutation Test v2 Results
Source: Diagnostics (Basel). 2025 Apr 8;15(8):948. doi: 10.3390/diagnostics15080948 (PMC12025441; doi:10.3390/diagnostics15080948)
Supplement: Supplementary file 1 [file diagnostics-15-00948-s001.zip › diagnostics-3496873-supplementary.pdf]

Supplementary Materials

Supplementary Table S1. Specific EGFR primers used for Sanger sequencing.

Primer	Sequence
EGFR Exon 18F	5'-CAAATGAGCTGGCAAGTGCCGTGTC-3'
EGFR Exon 18R	5'-CCAAACACTCAGTGAAACAAAGAG-3'
EGFR Exon 19F	5'-GCAATATCAGCCTTAGGTGCGGCTC-3'
EGFR Exon 19R	5'-AGCAGGGTCTAGAGCAGAGC-3'
EGFR Exon 20F	5'-CCATGAGTACGTATTTTGAAACTC-3'
EGFR Exon 20R	5'-CATATCCCCATGGCAAACTCTTGC-3'
EGFR Exon 21F	5'-CAGCCATAAGTCCTCGACGTGG-3'
EGFR Exon 21R	5'-CATCCTCCCCTGCATGTGTTAAAC-3'
